# β-Ionone Attenuates Dexamethasone-Induced Suppression of Collagen and Hyaluronic Acid Synthesis in Human Dermal Fibroblasts

**DOI:** 10.3390/biom11050619

**Published:** 2021-04-21

**Authors:** Dabin Choi, Wesuk Kang, Soyoon Park, Bomin Son, Taesun Park

**Affiliations:** Department of Food and Nutrition, BK21 FOUR, Yonsei University, 50 Yonsei-ro, Seodaemun-gu, Seoul 120-749, Korea; vin1411@naver.com (D.C.); wesuk42@naver.com (W.K.); thdbs1201@naver.com (S.P.); mim1110@naver.com (B.S.)

**Keywords:** β-ionone, glucocorticoid, collagen, hyaluronic acid, stress

## Abstract

Stress is a major contributing factor of skin aging, which is clinically characterized by wrinkles, loss of elasticity, and dryness. In particular, glucocorticoids are generally considered key hormones for promoting stress-induced skin aging through binding to glucocorticoid receptors (GRs). In this work, we aimed to investigate whether β-ionone (a compound occurring in various foods such as carrots and almonds) attenuates dexamethasone-induced suppression of collagen and hyaluronic acid synthesis in human dermal fibroblasts, and to explore the mechanisms involved. We found that β-ionone promoted collagen production dose-dependently and increased mRNA expression levels, including collagen type I α 1 chain (*COL1A1*) and *COL1A2* in dexamethasone-treated human dermal fibroblasts. It also raised hyaluronic acid synthase mRNA expression and hyaluronic acid levels. Notably, β-ionone inhibited cortisol binding to GR, subsequent dexamethasone-induced GR signaling, and the expression of several GR target genes. Our results reveal the strong potential of β-ionone for preventing stress-induced skin aging and suggest that its effects are related to the inhibition of GR signaling in human dermal fibroblasts.

## 1. Introduction

Human skin is mostly composed of a dense collagen-rich extracellular matrix (ECM) that provides mechanical and structural support. Hyaluronic acid, which is embedded between bundles of collagen fibers, binds water and contributes to skin hydration [1,2,3]. Nonetheless, during skin aging induced by various factors, such as stress, ultraviolet light, and smoking, the ECM undergoes progressive destruction, mainly resulting from collagen breakdown and hyaluronic acid degradation [4,5,6,7,8,9]. In particular, stress is widely accepted as an inevitable part of daily life because it can detrimentally affect individuals of all ages. Notably, even a short period (a few days–weeks) of topical stress hormone (glucocorticoid) treatment is sufficient to induce a marked reduction in collagen and hyaluronic acid levels in the skin of healthy volunteers [10,11]. In rodents, daily rotational stress induces skin aging, as evidenced by reduced collagen deposition accompanied by the downregulation of collagen mRNA [12].

Glucocorticoids are generally considered stress hormones, and each of them binds to a glucocorticoid receptor (GR); the resulting complex can mediate most stress responses through the transcriptional regulation of various genes. The glucocorticoid–GR complex typically regulates the transcription of target genes by binding to glucocorticoid-responsive elements (GREs) located in the promoter sequences of these genes and can exert some actions by interfering with other transcription factors such as SMAD [13,14,15,16]. Although a consensus has not been reached regarding the exact mechanism, these GR target genes and the regulation of other transcription factors by GR are regarded as the main mechanisms responsible for glucocorticoid-induced ECM degradation [17,18,19,20].

Despite advances in the understanding of the molecular mechanisms behind glucocorticoid-induced skin aging, little progress has been made in the development of highly potent compounds against this devastating condition. Previously, in our in-house phytochemical library, we tried to reveal substances that can increase the procollagen synthesis in the human-dermal-fibroblast model of dexamethasone (synthetic glucocorticoid)-induced skin aging. In that study, we found β-ionone (C_13_H_20_O)—an aroma compound present in various foods such as carrots, raspberries, roasted almonds, tea, tangelos, and tomatoes—to be a good candidate molecule for further investigation. β-ionone has the generally recognized as safe (GRAS) status. It is also approved as a flavoring ingredient in food by the Food and Drug Administration (FDA). Previous studies revealed that this compound has antioxidant capacity and anti-proliferative and pro-apoptotic effects on several cell lines, such as human lung epithelial cells and leucocytes [21,22,23,24,25,26,27]. In the present work, we aimed to investigate whether β-ionone attenuates the dexamethasone-induced suppression of collagen and hyaluronic acid synthesis, and we delineated the mechanisms involved.

## 2. Materials and Methods

### 2.1. Cell Culture

Human dermal fibroblasts (Hs68 cells), purchased from the American Type Culture Collection (Manassas, VA, USA), were cultured at 37 °C and 5% CO_2_ in 10% fetal bovine serum (FBS; Gibco, Eggenstein, Germany) and 1% streptomycin–penicillin solution (Gibco) supplemented with high-glucose Dulbecco’s Modified Eagle’s Medium (Gibco). After reaching 80% confluency, the cells were subcultured and used for further experiments.

### 2.2. Cell Viability Assay

Cell viability was assessed using the water soluble tetrazolium salt −1 (WST-1) assay (Sigma-Aldrich, Seoul, Korea). Hs68 cells (10^4^/well) were plated in a 96-well plate and cultured for 24 h. Then, the cells were rinsed with phosphate-buffered saline (PBS; Welgene, Daegu, Korea) and incubated with serum-free medium containing either vehicle (dimethyl sulfoxide (DMSO); Sigma-Aldrich) or 12.5–200 µM of β-ionone (Sigma-Aldrich) for 72 h. To evaluate the impact of β-ionone on cell viability in the presence of dexamethasone, the cells were cultured with either vehicle or 12.5–200 µM of β-ionone in the presence of 1 µM dexamethasone (Sigma-Aldrich) in serum-free medium for 72 h. After that, the medium was supplemented with 10% WST-1 for a 2 h incubation, followed by spectrophotometric measurement. The cleavage of WST-1 to formazan was detected at 450 nm by a microplate reader (M200; Tecan, Männedorf, Switzerland).

### 2.3. Measurement of Type I Procollagen and Hyaluronic Acid

Hs68 cells (6 × 10^4^/well) were plated in a 24-well plate and cultured for 24 h. The cells were then rinsed with PBS and serum-free medium was added, containing dexamethasone (1 µM) and either vehicle or various concentrations (12.5–50 µM) of β-ionone for a 24 h incubation. The culture medium was collected, and the production of type I collagen was assessed by measuring the procollagen type I C-peptide content in the culture supernatant using the enzyme-linked immunosorbent assay (ELISA) kits for type I procollagen (Takara, Shiga, Japan), conforming to the manufacturer’s protocols. The concentrations of hyaluronic acid in the culture supernatants were determined by an ELISA kit for hyaluronan (R&D Systems, Minneapolis, MN, USA), conforming to the manufacturer’s protocols. The data on hyaluronic acid and type I procollagen levels were normalized to the total protein concentration measured by the Bradford Assay (Bio-Rad, Hercules, CA, USA), with bovine serum albumin (BSA; LPS solution, Daejeon, Korea) as a standard.

### 2.4. Quantitative PCR

Hs68 cells (2 × 10^6^/well) were seeded in 90 mm cell culture dishes and cultured for 24 h. Next, the cells were rinsed with PBS and covered with serum-free medium containing 1 µM of dexamethasone and either vehicle or 50 µM of β-ionone for a 12 h incubation. After this treatment, total RNA samples were extracted from the cells using TRIzol reagent (Invitrogen, Carlsbad, CA, USA) and quantified using a NanoDrop spectrophotometer (M200; Tecan). After that, an equal amount of each RNA sample was subjected to cDNA synthesis with SuperScript IV Reverse Transcriptase (Invitrogen), according to the manufacturer’s instructions. Quantitative PCR was carried out in a final volume of 20 μL (comprising 10 μL of the Universal SYBR Green Supermix (Bio-Rad), 0.6 μM of each primer, and 50 ng of template cDNA) using a CFX Real-Time System (Bio-Rad). Glyceraldehyde-3-phosphate dehydrogenase (*GAPDH*) served as a reference gene for the normalization of gene expression. Primer sequences used for each gene are listed in Supplementary Table S1.

### 2.5. GR Competitor Assay

The binding of β-ionone to purified GR was assessed with the PolarScreen GR Competitor Assay Kit (Thermo Fisher Scientific, Bremen, Germany), following to the manufacturer’s instructions. GR was added to a glucocorticoid ligand, with or without various concentrations (10^−8^–10^−2^ M) of β-ionone and competitor test compound, in a 96-well plate. Since glucocorticoid ligand is itself fluorescent, if β-ionone competed with the glucocorticoid ligand, the glucocorticoid ligand/GR complex would not form and glucocorticoid ligand would rotate rapidly, resulting in a low polarization value. The decrease in the polarization value was used to determine the relative affinity of the test compounds for GR. Fluorescence polarization was determined using a FlexStation 3 microplate reader (Molecular Devices, Sunnydale, CA, USA) at 531 nm excitation wavelength and 595 nm emission wavelength, and the half-maximal inhibitory concentration (IC_50_) was calculated from the competition curve.

### 2.6. Western Blotting

Hs68 cells (2 × 10^6^/well) were seeded in 90 mm cell culture dishes and cultivated for 24 h. All serum-containing medium was removed and changed with serum-free medium. Then, the cells were cultured with either vehicle or 50 µM of β-ionone in the presence of 1 µM of dexamethasone for 30 min. After that, the cells were harvested and lysed using a protein extraction solution (PRO-PREP; iNtRON, Seoul, Korea). After centrifugation at 13,000× *g* for 20 min at 3 °C, proteins in the lysates were separated by sodium dodecyl sulfate polyacrylamide gel electrophoresis and transferred to a nitrocellulose membrane (Whatman, Dassel, Germany). The membranes were incubated for 2 h in tris-buffered saline, containing 5% BSA and 0.05% Tween 20, and were then incubated with primary antibodies at 4 °C overnight. The primary antibodies against the following proteins were obtained from Abcam (Cambridge, U.K.): GR (ab183127) and Ser211-phosphorylated GR (p-GR; ab55189). The primary antibody against GAPDH (#2118) was acquired from Cell Signaling (Danvers, MA, USA). Next, the membranes were probed for 1 h with an anti-rabbit IgG-linked secondary antibody (Santa Cruz Biotechnology, Santa Cruz, CA, USA). Reaction products were visualized with an electrochemiluminescence detection reagent (Bio-Rad). Relative band densities were quantified by densitometry using the Quantity One software (v4.6.2; Bio-Rad). GAPDH served as a loading control.

### 2.7. Statistical Analysis

Each set of experiments was performed three times independently, and the values were represented as mean ± standard error of mean (SEM). Differences between groups were tested using SPSS 25 software (SPSS; Chicago, IL, USA) by unpaired Student’s *t*-test. Data with *p* < 0.05 were considered statistically significant.

## 3. Results

### 3.1. β-Ionone Has no Effect on the Viability of Dexamethasone-Untreated and Dexamethasone-Treated Human Dermal Fibroblasts

**Figure 1:**
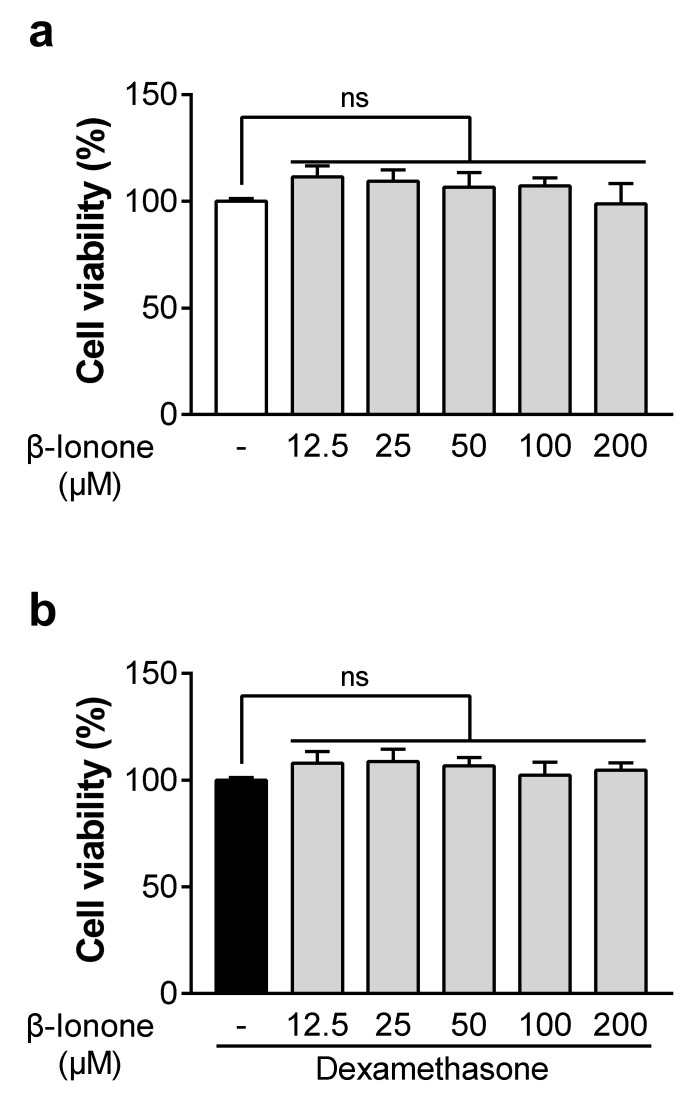
(**a**,**b**) Effects of β-ionone on the viability of dexamethasone-untreated and dexamethasone-treated human dermal fibroblasts. The cells were treated for 72 h with either vehicle (dimethyl sulfoxide (DMSO); shown as “-“) or different doses of β-ionone (12.5, 25, 50, 100, or 200 µM) with or without dexamethasone (1 µM). Cell viability was assessed by the WST-1 assay. Values are shown as mean ± standard error of the mean (SEM) of three experiments.

To determine whether β-ionone affects cell viability, we performed the WST-1 assay on human dermal fibroblasts that were either untreated or treated with dexamethasone. At concentrations up to 200 μM, β-ionone had no influence on the viability of dexamethasone-untreated and dexamethasone-treated human dermal fibroblasts (Figure 1a,b).

### 3.2. β-Ionone Attenuates Dexamethasone-Induced Suppression of Collagen Synthesis in Human Dermal Fibroblasts

**Figure 2:**
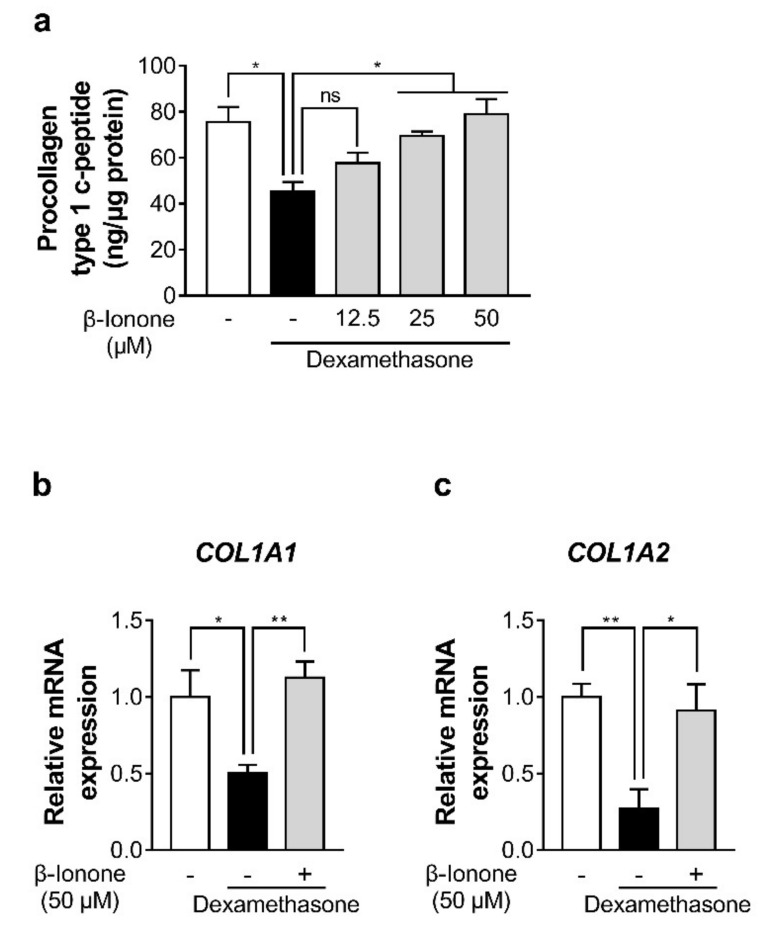
Impact of β-ionone on collagen synthesis in human dermal fibroblasts. The cells were treated with either vehicle (shown as “-”) or three doses of β-ionone (12.5, 25, or 50 µM (shown as “+”)) and dexamethasone (1 µM) for 24 h. (**a**) The procollagen type I C-peptide content was measured in the culture supernatants of the dermal fibroblasts, and (**b**,**c**) the gene expression levels of collagen type I α 1 chain (*COL1A1*) and *COL1A2* were quantitated in the cells. Values are shown as mean ± standard error of the mean (SEM) of three experiments. Statistical significance is expressed as follows: * *p* < 0.05, ** *p* < 0.01.

To investigate whether β-ionone can enhance collagen production in dexamethasone-treated human dermal fibroblasts, we quantified procollagen type I C-peptide in the culture medium and gene expression levels of collagen type I α 1 chain (*COL1A1*) and *COL1A2* in human dermal fibroblasts. Dexamethasone significantly decreased type I procollagen production in comparison with control cells. In contrast, β-ionone enhanced the production of type I procollagen in a dose-related manner (Figure 2a). Consistent with this result, β-ionone significantly increased the gene expression levels of *COL1A1* and *COL1A2* in the dexamethasone-treated human dermal fibroblasts (Figure 2b). β-ionone treatment had no effect on the collagen synthesis in the basal model (without dexamethasone treatment) (Supplementary Figure S1).

### 3.3. β-Ionone Attenuates Dexamethasone-Induced Suppression of Hyaluronic Acid Synthesis in Human Dermal Fibroblasts

**Figure 3:**
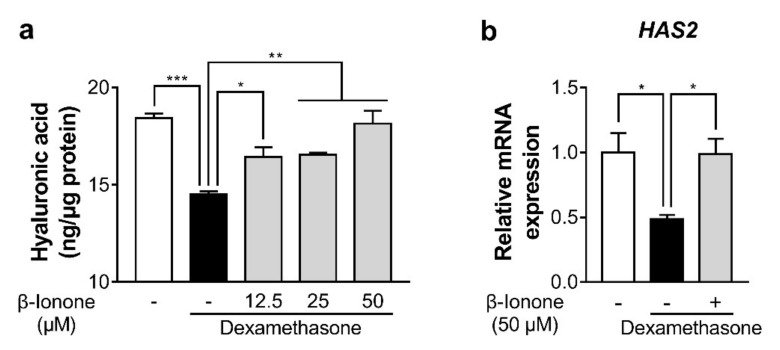
Effect of β-ionone on hyaluronic acid synthesis in human dermal fibroblasts. The cells were cultured with either vehicle (shown as “-”) or three concentrations of β-ionone (12.5, 25, or 50 µM (shown as “+”)) and dexamethasone (1 µM) for 24 h. (**a**) Hyaluronic acid content was measured in the culture medium of dermal fibroblasts, and (**b**) mRNA expression levels of hyaluronic acid synthase 2 (*HAS2*) were determined in the cells. Values are shown as mean ± standard error of the mean (SEM) of three experiments. Statistical significance is expressed as follows: * *p* < 0.05, ** *p* < 0.01, *** *p* < 0.001.

To test whether β-ionone promotes hyaluronic acid synthesis in dexamethasone-treated human dermal fibroblasts, we measured hyaluronic acid content in the culture supernatant and hyaluronic acid synthase 2 (*HAS2*) gene expression in the human dermal fibroblasts. Dexamethasone significantly reduced hyaluronic acid content, and β-ionone significantly reversed the dexamethasone-induced decrease in hyaluronic acid production in a dose-dependent manner (Figure 3a). Furthermore, β-ionone significantly increased mRNA expression of *HAS2* in dexamethasone-treated human dermal fibroblasts (Figure 3b).

### 3.4. β-Ionone Inhibits Glucocorticoid Binding to GR

**Figure 4:**
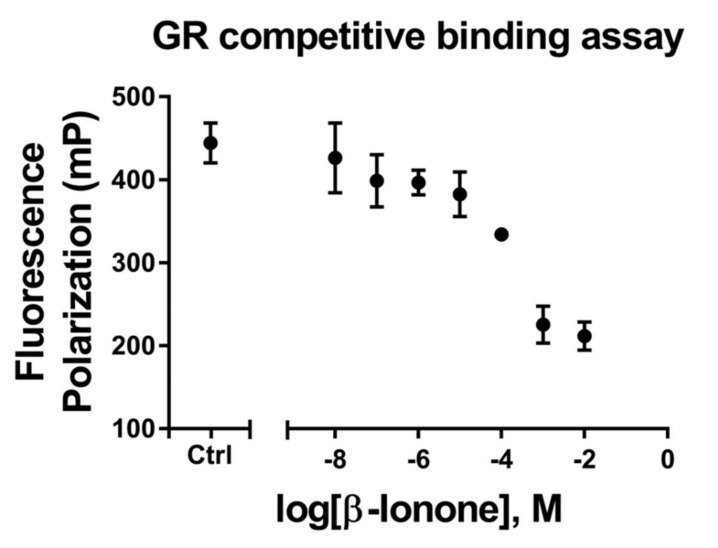
The inhibitory influence of β-ionone on glucocorticoid binding to glucocorticoid receptors (GRs). The human-GR–fluormone complex was incubated with either vehicle or various concentrations of β-ionone (10^−8^–10^−2^ M) for 2 h, and fluorescence polarization was measured. Values are shown as mean ± standard error of the mean (SEM) of three experiments.

To determine whether β-ionone inhibits glucocorticoid binding to GR, we performed a fluorescence polarization assay using full-length GR, a glucocorticoid fluorescent ligand, and β-ionone as a competitor. The binding of the glucocorticoid fluorescent ligand was inhibited in a dose-related manner by β-ionone with an IC_50_ of 66.07 μM (Figure 4).

### 3.5. β-Ionone Inhibits Dexamethasone-Induced GR Signaling in Human Dermal Fibroblasts

**Figure 5:**
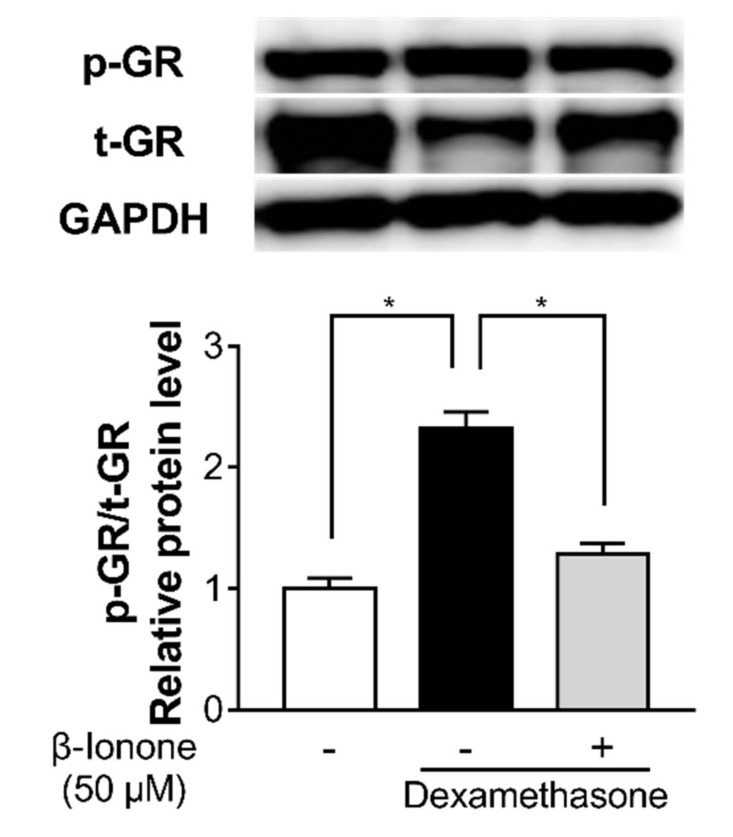
Effect of β-ionone on GR signaling. Human dermal fibroblasts were treated with either vehicle (shown as “-”) or β-ionone (shown as “+”) as well as dexamethasone for 30 min. Whole-cell protein extracts were prepared and analyzed by Western blotting. The levels of phosphorylated GR and total GR were normalized to the level of GAPDH, and then the ratio of phosphorylated/total GR was calculated. The ratio of phosphorylated GR to total GR is presented (p-GR/t-GR). Values are shown as mean ± standard error of the mean (SEM) of three experiments. Statistical significance is expressed as follows: * *p* < 0.05.

To investigate whether β-ionone inhibits dexamethasone-induced GR signaling, we estimated GR phosphorylation. To evaluate changes in the GR phosphorylation, the relative level of phosphorylated/total GR was calculated after GAPDH normalization based on a densitometry analysis of these protein bands. Dexamethasone induced GR phosphorylation in the human dermal fibroblasts; in contrast, β-ionone significantly inhibited the dexamethasone-induced phosphorylation of GR in the human dermal fibroblasts (Figure 5).

### 3.6. β-Ionone Suppresses Dexamethasone-Induced GR target genes in Human Dermal Fibroblasts

**Figure 6:**
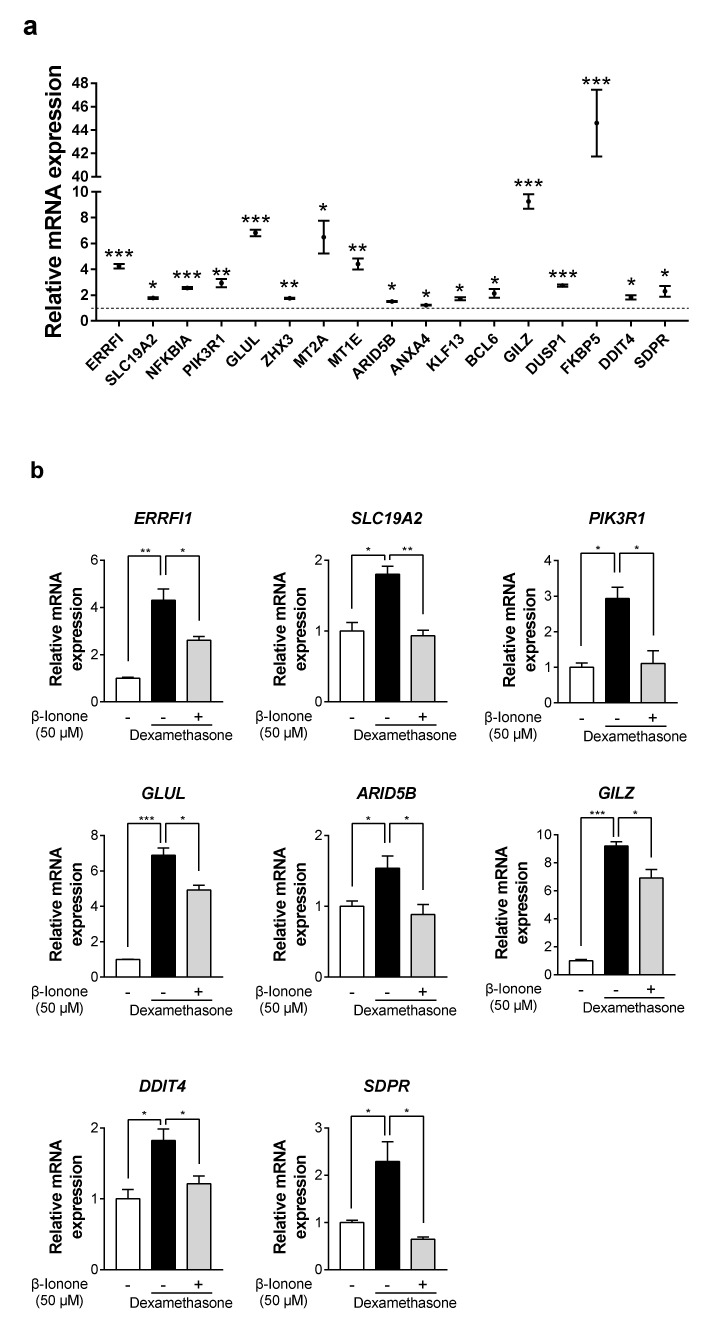
β-ionone suppresses dexamethasone-induced GR target genes in human dermal fibroblasts. Human dermal fibroblasts were treated with either vehicle (shown as “-”) or β-ionone (shown as “+”) as well as dexamethasone for 12 h. Total RNA samples were prepared and analyzed by quantitative RT-PCR. (**a**,**b**) The mRNA expression of well-known glucocorticoid-responsive genes (ErbB receptor feedback inhibitor 1, *ERRFI1*; solute carrier family 19 member 2, *SLC19A2*; nuclear factor kappa B inhibitor alpha, *NFKBIA*; phosphoinositide-3-kinase regulatory subunit 1, *PIK3R1*; glutamate-ammonia ligase, *GLUL*; zinc fingers and homeoboxes 3, *ZHX3*; metallothionein 2A, *MT2A*; metallothionein 1E, *MT1E*; AT-rich interaction domain 5b, *ARID5B*; annexin A4. *ANXA4*; kruppel-like factor 13, *KLF13*; B-cell lymphoma 6, *BCL6*; glucocorticoid-induced leucine zipper, *GILZ*; dual specificity phosphatase 1, *DUSP1*; FK506 binding protein 5, *FKBP5*; DNA damage inducible transcript 4, *DDIT4*; and serum deprivation-response protein, *SDPR*) was evaluated. Values are shown as mean ± standard error of the mean (SEM) of three experiments. Statistical significance is expressed as follows: * *p* < 0.05, ** *p* < 0.01, *** *p* < 0.001.

**Figure 7:**
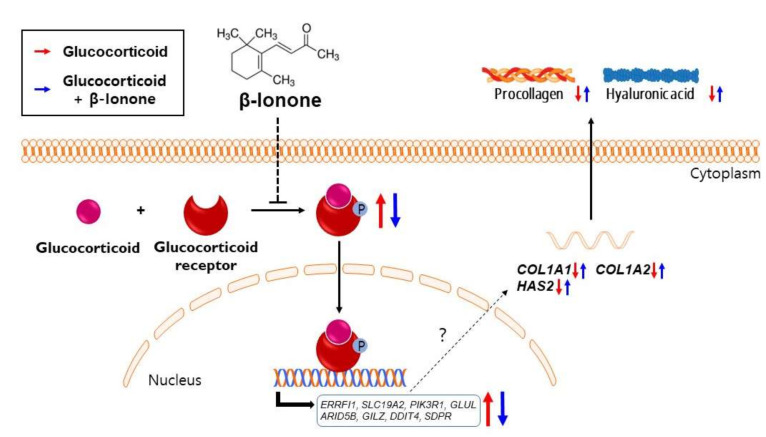
A suggested mechanism by which β-ionone attenuates glucocorticoid-induced suppression of collagen and hyaluronic acid synthesis in human dermal fibroblasts. As shown in a schematic illustration, the inhibition of glucocorticoid binding to its receptor by β-ionone suppresses the glucocorticoid-induced GR signaling and subsequent collagen and hyaluronic acid degradation. The detailed molecular mechanism of how GR transcription factor regulates collagen and hyaluronic acid needs further investigation.

To explore the mechanism underlying the beneficial effects of β-ionone, we selected 63 well-known GR target gene candidates based on the results of previous target gene prediction analysis [28,29,30,31,32,33]. The mRNA expression of 17 GR target genes was significantly upregulated by dexamethasone in the human dermal fibroblasts (Figure 6a); among these, the mRNA expression of eight genes (including ErbB receptor feedback inhibitor 1 (*ERRFI1*), solute carrier family 19 member 2 (*SLC19A2*), phosphoinositide-3-kinase regulatory subunit 1 (*PIK3R1*), glutamate-ammonia ligase (*GLUL*), AT-rich interaction domain 5b (*ARID5B*), glucocorticoid-induced leucine zipper (*GILZ*), DNA damage inducible transcript 4 (*DDIT4*), and serum deprivation-response protein (*SDPR*) was significantly suppressed by the treatment with β-ionone in dexamethasone-treated human dermal fibroblasts (Figure 6b). Taken together, the inhibition of glucocorticoid binding to receptors is a possible mechanism for the anti-skin-aging effects of β-ionone (Figure 7).

### 3.7. β-Ionone Has No Effect on the Expression of pro-Inflammatory Genes in Human Dermal Fibroblasts

To examine whether β-ionone inhibits the anti-inflammatory effects of dexamethasone, we measured the expression of two major pro-inflammatory genes, including interleukin 1 beta (*IL1B*) and interleukin 6 (*IL6*). The mRNA expression of these genes was significantly decreased by the treatment with dexamethasone, but β-ionone did not affect the expression of *IL1B* and *IL6* in either dexamethasone-treated or -untreated human dermal fibroblasts (Supplementary Figure S2a,b).

## 4. Discussion

Physiological stress triggers the release of corticotropin-releasing factor (CRF) from the hypothalamus, thereby causing the anterior pituitary to stimulate the synthesis and release of adrenocorticotropic hormone (ACTH). The latter then stimulates the adrenal cortex to produce glucocorticoids [34,35]. Circulating glucocorticoids can directly affect their peripheral target tissues, including in the skin. Thus, dexamethasone (a synthetic glucocorticoid) has been extensively used to mimic physiological stress in in vitro systems [36,37,38]. In clinical studies, it was reported that the serum level of cortisol in adult participants under severe psychological stress increases up to about 1 μM [39,40,41]. For example, in a study by Sanjeev et al., the average level of serum cortisol in a group complaining about job-related stress was found to be as high as 1.13 μM (*n* = 41); whereas, in an unstressed control group, it was only 0.39 μM (*n* = 67) [40]. Therefore, for the present study, 1 μM was chosen as the optimal concentration of dexamethasone to mimic severe stressful conditions that can be encountered in daily life.

Collagen is the single most abundant component in the human body and constitutes approximately 25–35% of dry body weight. Although 28 types of collagen have been identified in humans, type I collagen comprises approximately 90% of total collagen in the skin [42,43]. Although hyaluronic acid makes up only ~0.1–0.3% of dry skin weight, it can bind up to 1000 times its weight of water [44,45,46]. Hyaluronic acid is produced in the plasma membrane by HAS1–3; HAS2 appears to be the dominant isoform in dermal fibroblasts because of the extent to which HAS2 upregulation correlates with an increase in hyaluronic acid secretion [47,48,49]. In the present study, β-ionone treatment attenuated dexamethasone-induced suppression of type I procollagen genes and of the *HAS2* gene and increased both procollagen and hyaluronic acid contents in human dermal fibroblasts.

In this in vitro study, β-ionone increased the secretion of procollagen and hyaluronic acid into the culture medium by dermal fibroblasts exposed to dexamethasone. It is commonly assumed that collagen and hyaluronic acid are independently responsible for skin elasticity and hydration, respectively, but it was recently suggested that there is a cross-talk between collagen and hyaluronic acid, and this cross-talk likely plays an essential role in the regulation of overall tissue structure [50,51,52]. Supporting this notion, a clinical study showed that the injection of hyaluronic acid promotes collagen production, thereby partially restoring dermal ECM components that are lost in aged human skin [53]. In another study, the degradation of collagen in response to ultraviolet B light exposure led to the downregulation of HAS and a subsequent reduction in hyaluronic acid levels with the help of bioactive collagen fragments [47]. If β-ionone acts in human skin tissues as it does in the human-dermal-fibroblast model, then increased levels of both collagen and hyaluronic acid may have synergistic antiaging effects under stressful conditions.

We demonstrated that β-ionone binds to GR as an antagonist and inhibits GR activation. Although there is no consensus regarding the ligand structure that can effectively antagonize GR, it has been proposed that the planar structure of the α-,β-unsaturated ketone (enone) ring, a common component of steroids, is essential for receptor binding [54,55]. Several synthetic enones have glucocorticoid-antagonistic properties, as evidenced by in vitro binding and functional assays [56]. β-ionone also contains an enone moiety, which, presumably, may contribute to the binding to GR. Recent studies have shown that β-ionone can bind to and stimulate olfactory receptor family 51 subfamily E member 2 (OR51E2), leading to the activation of various kinases, including protein kinase A (PKA) and protein kinase B (AKT), in an androgen-receptor-dependent and -independent manner [57,58,59]. Considering these kinases are thought to protect against skin aging, we cannot exclude the possibility that the anti-skin-aging effects of β-ionone partially occurs via OR51E2.

Glucocorticoid hormone is accepted as an important regulator of proliferation, differentiation, inflammation, and metabolism in skin [60,61,62,63]. Numerous studies have suggested that exposure to elevated glucocorticoid can be either beneficial or detrimental depending on the duration: as high levels of glucocorticoid are prolonged in the chronic phase, the various beneficial effects tend to disappear, and harmful effects become more evident [64,65,66,67]. β-ionone appears to mediate the beneficial effects in the skin of subjects with chronic stress conditions.

Although it has been more than three decades since glucocorticoid-induced skin aging was first described [9,68], how glucocorticoid signaling is involved in the skin aging is unfortunately still unclear; it seems to be contextually determined in a cell- and stimulus-specific manner [28,29,33,69]. In an attempt to explore the mechanism underlying the beneficial effects of β-ionone in dexamethasone-induced human dermal fibroblasts, we confirmed that β-ionone suppresses eight dexamethasone-induced GR target genes. Notably, ERRFI1, GILZ, PIK3R1, and DDIT4 have been reported as the negative regulators of PI3K/AKT/mTOR signaling, which can enhance the collagen and hyaluronic acid synthesis [70,71,72,73,74]. SDPR is known to be a calcium-independent phospholipid-binding protein, which is a substrate for protein kinase C (PKC) activation [75]. Activated PKC can interact with the mitogen-activated protein kinase pathways and in turn stimulate transcription factors, such as nuclear factor-kappa B and activator protein-1 in human fibroblasts. These transcriptional factors are known to promote the degradation of the ECM through upregulation of matrix metalloproteinases and cyclooxygenase-2 expression and repress collagen synthesis in human fibroblasts [76,77,78,79]. GLUL belongs to the glutamine synthetase family that rapidly converts glutamate to glutamine. Glutamate, not glutamine, serves as an important precursor for proline, which is the major component of collagen protein and plays key roles in collagen stability [80,81,82,83]. It would be intriguing to determine which of the GR target genes is mainly responsible for the beneficial effects of β-ionone on dexamethasone-treated human dermal fibroblasts in the future. Several studies have also reported that glucocorticoid-GR complex can have a beneficial effect by interfering with other transcription factors such as nuclear factor kappa B (NF-kB), which transcriptionally induces various pro-inflammatory genes [84,85,86]. We confirmed that β-ionone acts as a dissociated GR ligand: it competes with glucocorticoids for GR binding, but possibly not transrepression.

## 5. Conclusions

β-ionone markedly weakened dexamethasone-induced collagen and hyaluronic acid degradation; this phenomenon is probably related to the inhibition of GR. Although additional research is necessary to further evaluate the potential usefulness of β-ionone for the development of an anti-skin-aging agent applicable to cosmetics and food products, the current study highlights the possible benefits of β-ionone in the treatment of skin aging related to various stressful conditions. Moreover, our work provides insights into the molecular mechanisms underlying the effects of β-ionone in human dermal fibroblasts.

## Data Availability

All data generated or analyzed during this study are included in this published article.

## Supplementary Materials

The following are available online at https://www.mdpi.com/article/10.3390/biom11050619/s1, Figure S1: β-Ionone treatment has no effect on the collagen synthesis in the basal model, Figure S2: β-Ionone has no effect on the expression of pro-inflammatory genes in human dermal fibroblasts, Table S1: Primer sequences.
