# Correction: Choi et al. β-Ionone Attenuates Dexamethasone-Induced Suppression of Collagen and Hyaluronic Acid Synthesis in Human Dermal Fibroblasts. *Biomolecules* 2021, *11*, 619

**DOI:** 10.3390/biom14101337

**Published:** 2024-10-21

**Authors:** Dabin Choi, Wesuk Kang, Soyoon Park, Bomin Son, Taesun Park

**Affiliations:** Department of Food and Nutrition, BK21 FOUR, Yonsei University, 50 Yonsei-ro, Seodaemun-gu, Seoul 120-749, Republic of Korea; vin1411@naver.com (D.C.); wesuk42@naver.com (W.K.); thdbs1201@naver.com (S.P.); mim1110@naver.com (B.S.)

The authors would like to modify the Conflicts of Interest section of the published paper [1]. Their changes are below:

**Conflicts of Interest:** The authors would like to specify that the corresponding author, Taesun Park, holds several patents and is the CEO of a cosmetic company that commercializes products that contain similar ingredients to those that were investigated in the abovementioned article.

The authors apologize for any inconvenience caused and would like to state that their scientific conclusions are unaffected. This correction was approved by the Academic Editor. The original publication has also been updated.
