# Supplementary material for: β-Ionone Attenuates Dexamethasone-Induced Suppression of Collagen and Hyaluronic Acid Synthesis in Human Dermal Fibroblasts
Source: Biomolecules. 2021 Apr 21;11(5):619. doi: 10.3390/biom11050619 (PMC8143342; doi:10.3390/biom11050619)
Supplement: Supplementary file 1 [file biomolecules-11-00619-s001.zip › biomolecules-1107565-supplementary.pdf]

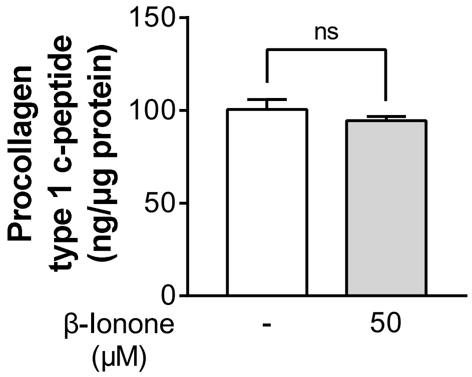

Supplementary Figure 1. β-Ionone treatment has no effect on the collagen synthesis in the basal model. The cells were treated with vehicle (dimethyl sulfoxide) or β-ionone (50 μM) for 24 h and the procollagen type I c-peptide content was measured in the culture supernatants of the dermal fibroblasts. Values are shown as mean ± standard error of the mean (SEM) of three experiments. Statistical significance is expressed as follows: ns, not significant (p > 0.05).

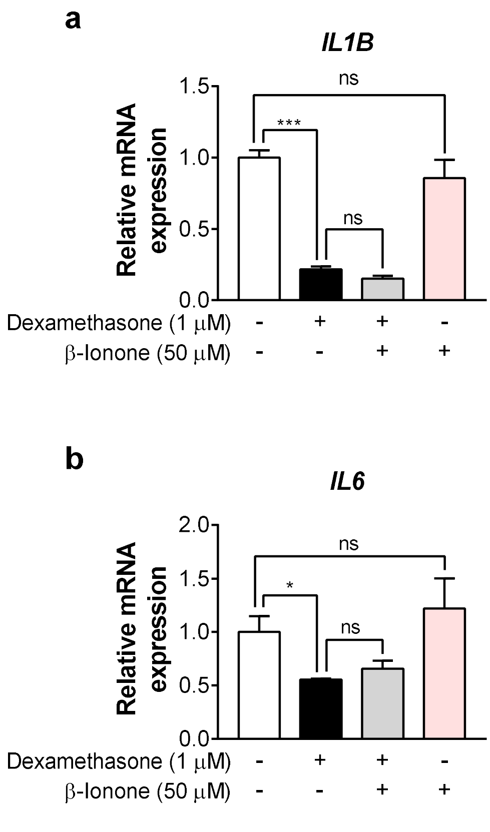

Supplementary Figure 2. β-Ionone has no effect on the expression of pro-inflammatory genes in human dermal fibroblasts. (**a**,**b**) The cells were treated with vehicle (DMSO), dexamethasone (1 μM) or β-ionone (50 μM) for 12 h and gene expression levels of interleukin 1 beta (IL1B) and IL6 were measured. Values are shown as mean ± standard error of the mean (SEM) of three experiments. Statistical significance is expressed as follows: ns, not significant (p > 0.05), *p < 0.05, ***p < 0.001.

Supplementary Table 1. Primer sequences.

Gene description	Sequence (5′→3′)
Annexin A4 (*ANXA4*)	F: ACCAGCAGCAATATGGACGG<br>R: TTCGGTTCCGGGAACAGAG
AT-rich interaction domain 5b (*ARID5B*)	F: GCAGTCACATGCTGTAGCTTTC<br>R: TCTTCTTTGTGGGCATGGTTTC
B-cell lymphoma 6 (*BCL6*)	F: GTCCTGCAGCAGTAAGAATGCCTG<br>R: GGCTGTTGAGCACGATGAACTTGT
Collagen type Ⅰ α 1 chain (*COL1A1*)	F: ACATGTTCAGCTTTGTGGACC<br>R: TGTACGCAGGTGATTGGTGG
Collagen type Ⅰ α 2 chain (*COL1A2*)	F: CGGACTTTGTTGCTGCTTGC<br>R: CAGCAAAGTTCCCACCGAGA
DNA damage inducible transcript 4 (*DDIT4*)	F: TGAGGATGAACACTTGTGTGC<br>R: CCAACTGGCTAGGCATCAGC
Dual specificity phosphatase 1 (*DUSP1*)	F: TGGAGGAAGGGTGTTTGTCC<br>R: CAAGGCAGATGGTGGCTGA
ErbB receptor feedback inhibitor 1 (*ERRFI1*)	F: GGCCTCACAGGTTTGGAGATG<br>R: TTCATCGGAGCAGATTTGGAAG
FK506 binding protein 5 (*FKBP5*)	F: GTCCAAAGCCTCAGAGTCGTTC<br>R: AGCCTTTCTCATTGGCACTGTC
Glyceraldehyde-3-phosphate dehydrogenase (*GAPDH*)	F: TCTGGAAAGCTGTGGCGTGA<br>R: TACTTGGCAGGTTTCTCCAGG
Glucocorticoid-induced leucine zipper (*GILZ*)	F: TCCTGTCTGAGCCCTGAAGAG<br>R: AGCCACTTACACCGCAGAAC
Glutamate-ammonia ligase (*GLUL*)	F: AGAAGAGCGGAGCGTGTGAG<br>R: CATGGTGGAAGGTGTTCTGGTC
Hyaluronic acid synthase 2 (*HAS2*)	F: GAGCAGCCCATTGAACCAGA<br>R: AGGAAGCGCAGAATTGGGAG
Interleukin 1 beta (*IL1B*)	F: GAGCTCGCCAGTGAAATGATGG<br>R: CTTGCTGTAGTGGTGGTCGG
Interleukin 6 (*IL6*)	F: TGCAATAACCACCCCTGACC<br>R: GTGCCCATGCTACATTTGCC
Kruppel-like factor 13 (*KLF13*)	F: ACGCCAACTTCCACCCAG<br>R: TTCGCTCAGCTTTCCTATTACC
Metallothionein 1E (*MT1E*)	F: CCCTTTGCTCGAAATGGA<br>R: GGGTTTGTGTCCCACGAG
Metallothionein 2A (*MT2A*)	F: GCTCCTGCAAATGCAAAGAGTG<br>R: CTTGTCCGACGCCCCTTTG
Nuclear factor kB inhibitor α (*NFKBIA*)	F: AGCACAAAGAGAGTGTCGC<br>R: CAGCGTTCATGGTTATGG
Phosphoinositide-3-kinase regulatory subunit 1 (*PIK3R1*)	F: AGCATTGGGACCTCACATTACACA<br>R: ACTGGAAACACAGTCCATGCACATA
Serum deprivation response protein (*SDPR*)	F: AGTCACGGTGCTCACGCTCC<br>R: GTTGCTGGTGGAGGCCTGGT
Solute carrier family 19 member 2 (*SLC19A2*)	F: AGCCAGACCGTCTCCTTGTA<br>R: TAGAGAGGGCCCACCACAC
Zinc fingers and homeoboxes 3 (*ZHX3*)	F: GGTGTCTGAGAACAGTGAGTCG<br>R: GCAGGCGGTTTCCCAGACTGG
